# The Sample, the Spectra and the Maths—The Critical Pillars in the Development of Robust and Sound Applications of Vibrational Spectroscopy

**DOI:** 10.3390/molecules25163674

**Published:** 2020-08-12

**Authors:** Daniel Cozzolino

**Affiliations:** 1 Centre for Nutrition and Food Sciences, Queensland Alliance for Agriculture and Food Innovation (QAAFI), The University of Queensland, Brisbane, Queensland 4072, Australia; d.cozzolino@uq.edu.au; 2 ARC Training Centre for Uniquely Australian Foods, Queensland Alliance for Agriculture and Food Innovation, The University of Queensland, Block 10, Level 1, 39 Kessels Rd, Coopers Plains Qld 4108, Australia

**Keywords:** vibrational spectroscopy, sampling, multivariate data analysis, error, validation

## Abstract

The last two decades have witnessed an increasing interest in the use of the so-called rapid analytical methods or high throughput techniques. Most of these applications reported the use of vibrational spectroscopy methods (near infrared (NIR), mid infrared (MIR), and Raman) in a wide range of samples (e.g., food ingredients and natural products). In these applications, the analytical method is integrated with a wide range of multivariate data analysis (MVA) techniques (e.g., pattern recognition, modelling techniques, calibration, etc.) to develop the target application. The availability of modern and inexpensive instrumentation together with the access to easy to use software is determining a steady growth in the number of uses of these technologies. This paper underlines and briefly discusses the three critical pillars—the sample (e.g., sampling, variability, etc.), the spectra and the mathematics (e.g., algorithms, pre-processing, data interpretation, etc.)—that support the development and implementation of vibrational spectroscopy applications.

## 1. Introduction

The last two decades have witnessed an increasing interest in the use of the so-called rapid analytical or high throughput techniques [1,2,3,4,5,6,7,8]. Most of these applications are based on the use of vibrational spectroscopy methods (near infrared (NIR), mid infrared (MIR), and Raman, visible (VIS)) in a wide range of samples (e.g., food ingredients, natural products, crops, animal and plant tissues, medical and pharmaceutical applications; etc.) [9,10,11,12,13]. The number of references using words such as “infrared”, “NIR”, “Raman”, “MIR”, “hyperspectral”, “green analytical methods”, “chemometrics” and “multivariate data analysis” [9,10,11,12,13,14,15,16,17], are evidence of this steady increase in the number of applications of these analytical methods.

In recent years, vibrational spectroscopy has been also considered for its potential as a high throughput phenotyping tool in both animals and plants, where novel applications related with plant breeding and selection, plant nutrition and physiology have been reported in the last 20 years [9,10,11,12,13,14,15]. More recently, vibrational spectroscopy (e.g., NIR, MIR, Raman and hyperspectral imaging systems) techniques have shown their ability to qualitatively (e.g., classifying, identifying, and monitoring) analyse several types of samples (e.g., wine, meat, coffee, condiments, etc.), targeting issues related with origin, traceability, and provenance of foods and food ingredients [9,10,11,12,13,14,15,16,17]. Concomitantly, recent developments in hardware (e.g., image techniques, optical sensors, handheld instrumentation, etc.) are adding new analytical possibilities to the potential users of these technologies, making them very attractive to be implemented in the whole food value chain (e.g., the addition and use of objective tools in blockchain and food traceability) [18,19,20].

Another field where vibrational spectroscopy demonstrated to have a great impact is in the so-called process analytical technologies (PAT) [21,22,23,24]. This approach has not only been used to collect chemical information about the process (e.g., spatial and temporal information) to monitor the composition of the product, but also to provide information about the process itself, such as yield, energy input, faults and quality assurance [21,22,23,24]. The implementation of vibrational spectroscopy based on the utilization of different type of sensors has provided a platform for process data analysis and process sensor technology [21,22,23,24]. The data collected by the sensor could be also utilised to provide useful information about other aspects of the process, such as occupational safety, sustainable protection of employees, plant safety, hazardous operating conditions, and to assure environmental protection, providing feedback about the conditions of the industry [21,22,23,24]. The incorporation of these technologies and the development of applications of PAT has increased the demands for a knowledge-based approach [21,22,23,24]. According to the researchers in the field, the integration of vibrational spectroscopy and other sensing techniques with multivariate data methods and techniques (MVA) caused PAT to boost the multidisciplinary approach within the industry and research, where the design of state-of-the-art sensors with high specificity and resolution have improved the amount of data collected and therefore the information in order to manage the data generated by these approaches [21,22,23,24].

This approach is not entirely strange to the industry where applications of these techniques also attracted an increase in interest from the pharmaceutical, food and beverage industries, etc. [9,10,11,12,13,14,15,16,17]. The main reasons for the increasing use of this approach might be due to the main advantages that these methods and techniques possess when compared with other routine analytical techniques or methods, such as the non-destructive nature of these technologies, minimal or no sample preparation, no chemical reagents required, easy and ready to use instrumentation, and availability of inexpensive and portable devices [9,10,11,12,13,14,15,16,17,18,19,20].

One of the main analytical advantages of rapid analytical methods or high throughput techniques is that they can evaluate/measure the biochemical and/or chemical characteristics of any given organic compound [9,10,11,12,13,14,15,16,17,18,19,20]. This might be possible as chemical bonds present in the sample vibrate at specific frequencies or wavelengths depending of their mass of the constituent atoms, the shape of the molecule, and the stiffness of the bonds [9,10,11,12,13,14,15,16,17,18,19,20]. Therefore, the amount and the frequency of the absorbed light and the total reflected or transmitted light can be used to infer the chemical composition of the sample. The chemical and/or physical information derived from the use of vibrational spectroscopic methods resides in the manifestation of peaks, band positions, intensities, and shapes [9,10,11,12,13,14,15,16,17,18,19,20].

In modern routine chemical analysis, scientist are often confronted with so much data that the essential information may be not readily evident [11,25,26,27,28,29,30,31]. This is the case with spectral data for which many different observations (peaks or wavelengths) have been collected during the analysis of the sample. Each different measurement can be thought of as a different dimension [11,25,26,27,28,29,30,31]. Therefore, the success of the application will be highly dependent on the integration with the most appropriate multivariate data analysis (MVA) method, such as pattern recognition and modelling techniques, to develop the target application [11,25,26,27,28,29,30,31].

The advances and developments in modern analytical instrumentation and, in particular, those observed in vibrational spectroscopy, have determined the increasing growth in the so called high-dimensional data, where both the number of measured variables and samples can be high, together with the high variety of data (e.g., multiple data sources are available) and high speed during the collection of the data [32,33,34]. Thus, the increasing use of vibrational spectroscopy has determined an increase in the volume, variety and velocity of data collected determining the so-called “big data” [32,33,34]. The generation and use of big data becomes the reality in the routine life of analytical chemists and every researcher [32,33,34]. Contradictory, although the time dedicated to analysing a single sample using vibrational spectroscopy has been reduced, the time dedicated to interpreting and mining the data has exponentially increased, depending on the dataset [32,33,34].

**Figure 1:**
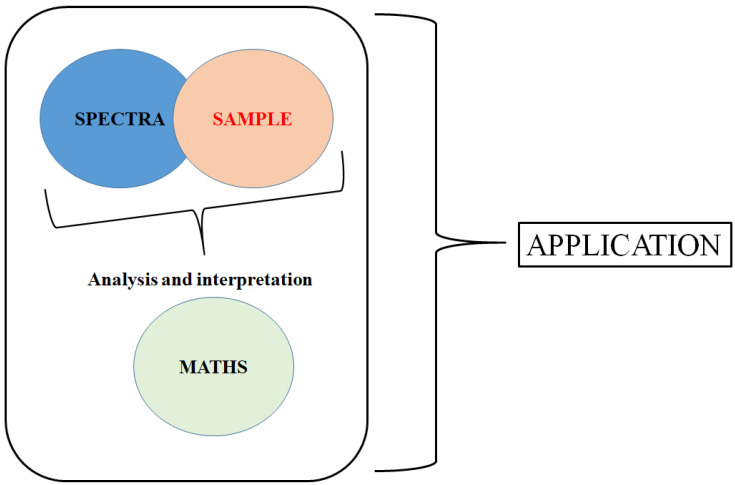
The integration or link between the sample, the method or technique and the mathematics during the development of applications based on vibrational spectroscopy.

Classical statistics are not able to handle the current increase in the volume of data generated with this approaches. In this context, the scope of MVA is wide where its applications are found in many fields and where the number of the so-called toolboxes or methods is diverse [11,25,26,27,28,29,30,31]. The integration of MVA into vibrational spectroscopy provides the means to move the analysis beyond the one-dimensional (univariate) space, revealing constituents or properties that are important through the various interferences and interactions in the matrix [11,25,26,27,28,29,30,31]. Today, many modern instrumental measurement techniques are multivariate and based on indirect measurements of the chemical and physical properties of the sample [11,25,26,27,28,29,30,31]. Figure 1 shows the theoretical and practical links between the sample, the method or technique and the mathematics during the development of an application.

Beyond the many advantages that the integration of vibrational spectroscopy with MVA offer, the ability of providing a holistic view of the system or sample analysed (e.g., fingerprint analysis) determines that these approaches are advantageous when compared with other analytical methods. In addition, the availability of modern and inexpensive instrumentation together with access to easy-to-use software is determining a steady growth in the number of applications of these technologies. Please note that this paper does not intend to be “another” review of multivariate data analysis and/or vibrational spectroscopy. The reader can find several excellent dedicated reviews already published in the scientific literature. Instead, the intention is to discuss and provide a guide of the main issues that can affect the successful implementation of these approaches.

Therefore, this paper underline and briefly discussed the three critical pillars—the sample (e.g., sampling, variability, etc.), the spectra and the mathematics (e.g., algorithms, pre-processing, data interpretation, etc.)—that support developments and implementations of vibrational spectroscopy applications.

## 2. The Source of Information—The Experiment and the Sample

### 2.1. The Theory of Sampling and Uncertainty

Regardless of all the care taken during sampling, the sample always differs in composition from the target intended [35,36,37,38]. Even the use of randomly replicated samples from the same target will differ among themselves, determining the so-called sampling uncertainty [35,36,37,38]. Understanding the uncertainty derived from both the sampling and the analysis will allow making rational decisions about a given process, classification or calibration results [35,36,37,38]. It is worth noting that the final application will be connected to making decisions about the target instead of about the sample [35,36,37,38].

Different authors have highlighted that one of the most important issues to be considered during sampling is related to how good the uncertainty depending on the purpose is [35,36,37,38]. One important issue to consider (and remember) is that the uncertainty of the measurement that arose from sampling is non-negligible [35,36,37,38,39]. This is even more significant when raw materials (e.g., food ingredients) and environmental samples (e.g., soil and water) are collected, where the uncertainty of the sampling exceeds the analytical contribution [35,36,37,38,39]. Therefore, the theory of sampling becomes highly relevant during the development of a given applications.

The theory of sampling (TOS) documents and details in a comprehensive means all aspects of the mechanical structure and chemical variation within a target in relation to the procedure for obtaining a primary sample from it [35,36,37,38,39]. Some of the main issues considered in the TOS are associated with the characteristics and/or properties of the target, including the size range of the particles comprising the target, the shapes of the particles, the compositional variation of the particles and the degree and style of the heterogeneity of the target, among others [35,36,37,38,39]. The method of collecting or extracting the sample and the degree of comminution/homogenisation/grinding at the different steps during the sampling process are important aspects included in the TOS [35,36,37,38,39]. All of these previously summarised issues and properties contributed to identifying the types of “error” of a given analysis or process [35,36,37,38,39].

The different sources and types of “errors” should be eliminated, and attention to detail will define the procedure or sampling protocol that will deliver the “correct” sample [35,36,37,38,39]. Researchers and practitioners in the field state that the interpretation of “correct” refers to “unbiased”, where sampling bias is avoided in the definition [35,36,37,38,39].

During the application of the TOS, it has been reported that sampling uncertainty is ignored and only the analytical uncertainty is considered [35,36,37,38,39]. The scientific literature in the field also suggested that the heterogeneity in the population and the ways of counteracting its adverse influence due to sampling/signal acquisition, sub-sampling and sample preparation/presentation processes, must be considered and evaluated before analysis [35,36,37,38,39].

In summary, the TOS is the main framework that must be used as a guide during meta-analysis of any application using vibrational spectroscopy [35,36,37,38,39]. It has been highlighted that the TOS emphasises the fundamental sampling principle (FSP), which states that all potential units from an original material must have an equal probability of being sampled in practice, and that samples are not altered in any way after sampling [35,36,37,38,39]. In the context of model development (e.g., calibration/validation and prediction), the main interactions between the sampling and the analysis (e.g., physical sampling), or the sampling and the on-line application, must be evaluated and understood in order to avoid inaccuracies and mistakes [35,36,37,38,39].

### 2.2. Samples

In any given application of vibrational spectroscopy, the sample itself plays an important role in defining the success of such application. However, the importance of both the sampling and the sample are usually overlooked. Two of the main characteristics or properties that define the success of a given application using vibrational spectroscopy are associated with both the perturbation and the observation of the sample [39,40,41,42,43,44,45,46]. The perturbation is usually associated with the experimental conditions used to develop the application (e.g., dry vs. wet sample, temperature, whole vs. powder, etc.) while the observations/samples are associated with the sampling protocol and the property to be measured (e.g., limit of detection, range in concentration, standard error of the laboratory, number of samples etc.) [39,40,41,42,43,44,45,46].

### 2.3. Sample Properties and Pre-Processing

Preparing, pre-processing (e.g., grinding and homogenisation) and selecting the samples to be incorporated into the application is not a trivial task [35,36,37,38,39]. During the process of preparing and selecting samples for analysis, several inconsistencies or errors can be added into the overall error of the method (e.g., multiplicative effects) [39,40,41,42,43,44,45,46]. For example, different pre-processing steps, such as drying and grinding of the sample, can contribute to significantly exacerbating the analytical error [39,40,41,42,43,44,45,46]. This kind of interaction between the perturbation and the observation can be observed in most of the applications using analytical methods, and they will define the success or lack thereof of the application based on the systematic error [39,40,41,42,43,44,45,46].

### 2.4. Sample Variability

Probably one of the main questions asked during the development of the application is associated with the selection of the most suitable sample to be used during calibration development [47]. It has been agreed by several researchers that samples used to build a given calibration model have to be selected from samples similar to those that will be analysed in the future [39,40,41,42,43,44,45,46,47,48]. In addition, the samples have to be exposed to the same pre-processing and handling steps adopted, and this should be maintained when future samples are incorporated into the calibration. Samples used in calibration must be sourced from a wide-range composition, or at least considering the expected range of the composition [39,40,41,42,43,44,45,46,47]. All sources of possible variation to be encountered in the future must be considered and/or incorporated into the sample set [39,40,41,42,43,44,45,46,47,48]. If samples are used to represent a process all potential variations in the system, factors such as temperature, changes in particle size, physical changes in the sample, and equipment should be incorporated [39,40,41,42,43,44,45,46,47]. When dealing with biological materials (e.g., plants, animal muscle or tissues), other variations must be evaluated, such as harvest time and type of tissue (e.g., type of muscle), among others [39,40,41,42,43,44,45,46,47,48].

However, the selection of samples is not an arbitrary task and demands care. For example, during calibration development, the aim is to obtain homogenous and representative samples well distributed along the dataset. If there are too many samples available, it is recommendable to choose samples in order to develop a well balance dataset. Although randomisation is the preferred method to select samples to be included into the calibration, a better approach will be the utilization of robust techniques based in either Mahalanobis and Euclidean distances or the Kennard–Stone algorithm [49,50]. Recently, the use kernel distances have been reported as a robust method to objectively select samples [49,50].

## 3. Collecting the Information—The Spectra

A wide range of analytical methods and techniques based on vibrational spectroscopy are available in the market nowadays (e.g., NIR, MIR, Raman, lab bench and handheld instrumentation, hyperspectral imaging etc.) [51,52,53]. All of these techniques have in common the fact that they generate large amounts of data. Munck and collaborators stated that most instruments based on vibrational spectroscopy are extensively used a black box devices for the estimation of chemical compositions based on calibrations [51,52,53]. Very few scientist are aware that black box technology can be expanded for the physical–chemical characterisation of spectra [51,52,53]. Please note that it is not the objective of this paper to provide a comprehensive and detailed description of the different vibrational methods used as rapid or high throughput methods [54,55,56,57,58,59,60,61,62,63,64]. More detailed information about the different methods and techniques available as well the different technical characteristics or properties of the commercial instrumentation available in the market can be found elsewhere [54,55,56,57,58,59,60,61,62,63,64].

The selection of the most appropriate measurement technique or sampling mode/method is also of importance. For example, the analysis of whole or powder samples (e.g., grains and forages) presents a much greater challenge than liquids (e.g., milk, wine, juice, etc.) when vibrational spectroscopy methods are used (e.g., NIR and ATR-MIR), since the measurements are generally made in the reflectance mode [39,40,41,42,43,44,45,46,47]. This is because reflectance measurements have lower energy collection efficiency than transmission measurements [39,40,41,42,43,44,45,46,47]. In addition, when using NIR reflectance measurements, light scattering efficiency is higher in the long wavelength region than at shorter wavelengths, which helps to offset the less efficient light collection [39,40,41,42,43,44,45,46,47]. Most of the applications of reflectance use the scattered light or energy from the sample, and they are used in the collection of NIR spectra [39,40,41,42,43,44,45,46,47]. The spectral characteristics of the sample can be also dramatically altered due to the particle size. Other properties that can have a large influence on the spectra might be related to suspended particles (e.g., fruit homogenates); the shape, size and orientation of particles in powders; and the sample thickness [39,40,41,42,43,44,45,46,47].

## 4. Analysing and Interpreting the Information—The Maths

The use of vibrational spectroscopy generates large amounts of data, allowing for the simultaneous analysis/measurement of several parameters, which provides a rapid and non-destructive quantification of major components in many organic substances [65,66,67,68,69,70,71,72,73,74,75,76,77,78]. The integration of vibrational spectroscopy methods with MVA has been the key for the success of the application of these techniques in many fields [65,66,67,68,69,70,71,72,73,74,75,76,77,78].

It has been stated (and sometimes is the believe by some of the users of MVA) that if the data already contain information, then any MVA method will succeed [35,36,37,38,39]. Unfortunately, the data are not as clean as expected when sampling and instrument noise and typing mistakes, among others have a greater impact where the use or pre-processing or any other correction does not improve the accuracy of the analytical results (e.g., inaccuracies can never be modelled) [35,36,37,38,39]. Therefore, a word of caution: MVA is not a “black box” or “push button” approach where the modelling will automatically do the rest [35,36,37,38,39].

### 4.1. Data Pre-Processing

Before starting with the analysis, interpretation and model developing, data pre-processing is a critical stage, as it affects the performance of the algorithms used and therefore the results (e.g., calibration and classification) [79,80,81,82,83]. Different methods and/or techniques for data pre-processing have been applied and developed specifically to different types of data and experimental designs [79,80,81,82,83]. For example, pre-processing of the spectra using the first and second derivatives, smoothing, multiple scatter correction (MSC), standard normal variate (SNV) and other normalization techniques were reported in most of the applications using vibrational spectroscopy [79,80,81,82,83]. Details about these pre-processing methods and techniques can be found in reviews by other authors [79,80,81,82,83].

### 4.2. Mistakes and Error during Analysis and Interpretation of the Data

The analysis of large-scale data is a challenging task (e.g., big data), not so much because the amount of data is large, but because large-scale measurement technologies possess high inherent variability [81,82,83,84]. Sources of this variability contribute to defining the systematic errors (bias) and the so-called stochastic effects (noise) [81,82,83,84,85,86]. Systematic effects influence all measurements in a similar manner [81,82,83,84,85,86]. Therefore, they can be eliminated or reduced using different data normalisation or pre-processing techniques [81,82,83,84,85,86]. However, stochastic effects cannot be corrected by pre-processing, but can be quantified, in particular by the application of repeated measurements (replicates) [81,82,83,84,85,86]. Depending on the modelling approach, further data manipulations might be necessary [81,82,83,84,85,86]. Ultimately, pre-processing techniques used to remove any irrelevant information that cannot be handled by the regression techniques [81,82,83,84,85,86]. Several pre-processing methods have been proposed and developed for this purpose and several references can be found elsewhere [81,82,83,84,85,86].

### 4.3. Algorithms Used to Develop Models

The use of MVA, unlike classic statistics, can also allow for the simultaneous analysis of multiple variables and takes collinearity into account (the variation in one variable, or group of variables, in terms of co-variation with other variables) [65,66,67,68,69,70,71,72,73,74,75,76,77,78,87,88,89,90,91,92,93,94,95]. The analysis can mathematically describe the co-variation (degree of association) between variables, or find a mathematical function (regression model) that calculates the values of the dependent variables from values of the measured (independent) variables [65,66,67,68,69,70,71,72,73,74,75,76,77,78,87,88,89,90,91,92,93,94,95].

The most commonly used data analysis algorithms for performing regression include partial least squares regression (PLS) and principal component regression (PCR) [65,66,67,68,69,70,71,72,73,74,75,76,77,78,87,88,89,90,91,92,93,94,95]. These regression methods are designed to avoid issues associated with noise and correlations (collinearity) in the data [65,66,67,68,69,70,71,72,73,74,75,76,77,78,87,88,89,90,91,92,93,94,95]. PLS has brought into the field an online analysis of plants for a variety of quality attributes [65,66,67,68,69,70,71,72,73,74,75,76,77,78,87,88,89,90,91,92,93,94,95]. Besides PLS and PCR regression, other multivariate data analysis methods have been applied either to explore datasets or to build calibration models, where principal component analysis (PCA); cluster analysis (CA); linear discriminant analysis (LDA) [65,66,67,68,69,70,71,72,73,74,75,76,77,78,87,88,89,90,91,92,93,94,95,96]; machine learning approaches, such as support vector machines, classification and regression (SVM) [97,98,99,100,101], artificial neural networks (ANN) and other non-linear techniques [102,103,104,105]; and pattern recognition methods are just few examples [63,64,65,66,67,68,69,70,71,72,73,74,75,76,85,86,87,88,89,90,91,92,93,94,95,96,97,98,99,100,101,102,103,104,105].

### 4.4. Validation

In practice, several applications of vibrational spectroscopy available in the scientific literature report the use of cross-validation as the main technique used to test the models [105,106,107,108,109,110,111,112,113,114,115,116,117,118,119,120,121]. One of the most important steps during the implementation of a calibration into a real-life situation is the process of verification, validation and required testing [105,106,107,108,109,110,111,112,113,114,115]. What appears to have improved in the last decades is the capability to manage the quality control, equation updates, and data analysis [105,106,107,108,109,110,111,112,113,114,115,122,123]. As mentioned above, in order to assess the accuracy of a calibration model and to avoid overfitting, validation procedures have to be applied; a calibration model without validation is nonsense [105,106,107,108,109,110,111,112,113,114,115]. Although in feasibility studies cross-validation can be the best practical method to demonstrate that a model can predict the measured property, the actual accuracy must be estimated with an appropriate test set or validation set [105,106,107,108,109,110,111,112,113,114,115]. For feasibility studies, different cross-validation techniques can be used [105,106,107,108,109,110,111,112,113,114,115,116,117,118,119,120,121,122,123,124]. For example, in leave-one-out cross-validation, one sample is removed from the dataset, and a calibration model is constructed for the remaining subset [104,105,106,107,108,109,110,111,112,113,114]. The removed samples are then utilised to calculate the prediction residual [105,106,107,108,109,110,111,112,113,114,115]. The process is repeated with other subsets until every sample has been left out once, and in the end, the variance of all prediction residuals is estimated. In multifold cross-validation, a well-defined number of samples (‘segment’) is left out instead of one [105,106,107,108,109,110,111,112,113,114,115]. In internal validation, the dataset is split into a calibration set and a validation set. Calibration models are determined to be robust when the prediction accuracy is relatively insensitive towards unknown changes of external factors [105,106,107,108,109,110,111,112,113,114,115,122,123,124].

A good validation method should include a dataset of a completely excluded set of samples (not included in cross-validation) sourced from a separate sample regime with separate analysis. An independent testing of the calibration models on an excluded validation set eludes several of the most frequent mistakes in MVA, such as model overfitting [105,106,107,108,109,110,111,112,113,114,115,120,121,122,123,124].

Validation of classification models (e.g., discrimination) derived from the application of hyperspectral imaging have their own challenges [105,117]. A recent tutorial revised the different validation methods used in hyperspectral imaging analysis [105,117]. One of the main issues encountered is related with the samples used to develop the models. If too many samples are used (e.g., oversampling), unconstrained bootstrap and k-fold cross-validation might yield inaccurate results, failing to provide a realistic estimate of the predictive performance of the model [105,117]. Factors that can have a large influence during the analysis might be related to the range of data points (e.g., wavenumbers) used, the size of the image, the distribution of pixels from the different classes in the image and the number of pixels included in the training set [117]. The authors of the tutorial indicated that better results were obtained when randomised samples were used to develop the calibration and validation datasets [117].

The development of discriminant models utilising image data acquired from a single sample is highly risky, as the models might not take into consideration the effect of several sources inducing variation in the IR signal (e.g., age, body mass index, collection dates, sample storage or instrument performance) [105,114]. Therefore, validation using an external validation set is necessary in order to avoid overoptimistic results [105,116,117]. Other validation methods have been proposed during the integration of discriminant approaches to hyperspectral image analysis [116,122,123,124]. A summary of these applications can be found in a review by Guaita and collaborators [116,122,123,124].

### 4.5. Data Interpretation

One of the main issues is the comparison of results from the literature is usually complicated by variations in the population size and structure with respect to the attribute of interest. It is therefore critical to report the standard deviation (SD) of the population for the attribute of interest [28,40,41,46,48,78,109,110,113]. In general, a range of statistics is required to be reported in order to compare different calibrations, including the coefficient of correlation (R), root mean square for the standard error in cross-validation (RMSECV), standard error of prediction (SEP), SD, the number of samples used, the number of outliers removed, and the number of principal components [28,40,41,46,48,78,109,110,113,116]. The report of marginal gains in the standard of cross-validation or prediction after the use of several pre-processing methods should be avoided. The same can be applied when different algorithms are used with no real improvements in the predictive ability of the models. A summary of the main statistics to be considered during calibration interpretation and reporting can be found in the report by Williams and collaborators [112].

Calibration models are often evaluated and/or reported using a combination of some of the statistics presented above. However, the sole interpretation and evaluation of statistics is not enough, and the loadings or coefficients of regression must be interpreted in the context of the property or the measured chemical analyte [28,40,41,46,50,78,109,112,113]. For example, if a calibration was developed to measure or predict protein, it is expected that wavelengths or frequencies that contain information about the N–H bonds will be prevalent. In real-life applications of vibrational spectroscopy, the calibration or model must be judged or considered in relation to their fit-for-purpose criterion [28,40,41,46,50,78,109,112,113].

## 5. Outliers, Overfitting and Underfitting

Typing errors; file transfer; interface errors; sensor malfunctions; and fouling, bad or incorrect sampling or sample presentation of the instrument, among other factors [117,118,119,120,121,122,123,124], may induce outliers. Samples can be considered as outliers according to the spectra, reference, or both [117,118,119,120,121,122,123,124]. During calibration development, outliers can be visualised using a principal component (PCA) scores plot [117,118,119,120,121,122,123,124]. Outlier samples should be kept during the initial steps of calibration until further investigation into their origin, and only the sample outliers that have an effect on the regression model are to be removed [117,118,119,120,121,122,123,124]. In any case, excessive pruning of the dataset for outliers should be avoided [117,118,119,120,121,122,123,124].

During the application of any of the MVA techniques presented above, it is important to select the appropriate number of components or latent variables (optimization) [117,118,119,120]. In this process, there is a delicate balance: if too many are used, there is too much redundancy in the independent variables used during the development of the model, causing the model to become overfitted [117,118,119,120]. In this case, the calibration model will be very dependent on the dataset and might provide poor prediction results [117,118,119,120,121,122,123,124]. On the other hand, using too few components will cause underfitting and the model will not be large enough to capture the variability in the data [117,118,119,120]. This “fitting” effect is strongly dependent on the number of samples used to develop the model and, in general, more samples give rise to more accurate predictions [117,118,119,120,121,122,123,124].

Overall, the use of MVA has the risk of overfitting (over-parameterization) determining a potential increase in the risk of false discovery [121]. Overfitting can be reduced during exploratory applications of vibrational spectroscopy by the use of rank optimization (e.g., based on pragmatic cross-validation), or by the use of double cross-validation (cross-model validation) [121]. These approaches, although not ideal, can be used until large, representative and independent test sets are obtained [121].

**Figure 2:**
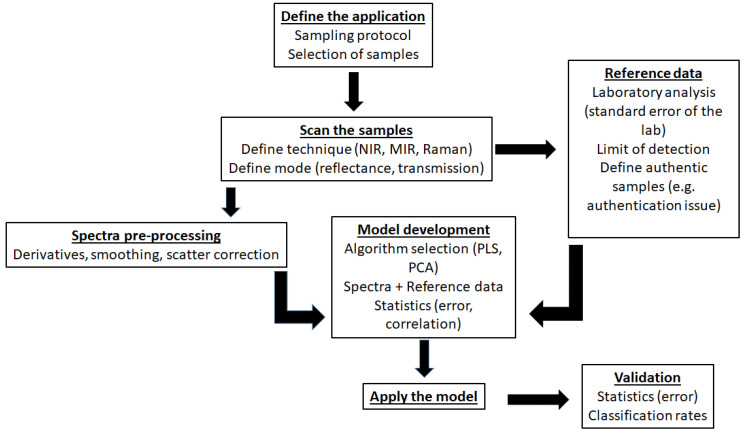
Steps needed to develop an application combining the sample, the spectra and the reference data.

The steps needed to develop an application combining the sample, the spectra and the reference data are summarised in Figure 2.

## 6. Concluding Remarks

The integration of vibrational spectroscopy with MVA to develop analytical applications (e.g., calibration and classification) can be considered by the non-expert purely as a mathematical or statistical exercise. This, however, could not be further from the truth—calibration development is a complex process that implies the understanding of a system created by the sample and its inherent characteristics (e.g., physical and chemical properties, variability, origin, pre-processing, etc.), the origin of the spectra (e.g., instrument characteristics, sample collection mode, etc.) and all the aspects of the multivariate data analysis (e.g., pre-processing, selection of samples for calibration and validation, linear and non-linear algorithms, outliers, etc).

**Figure 3:**
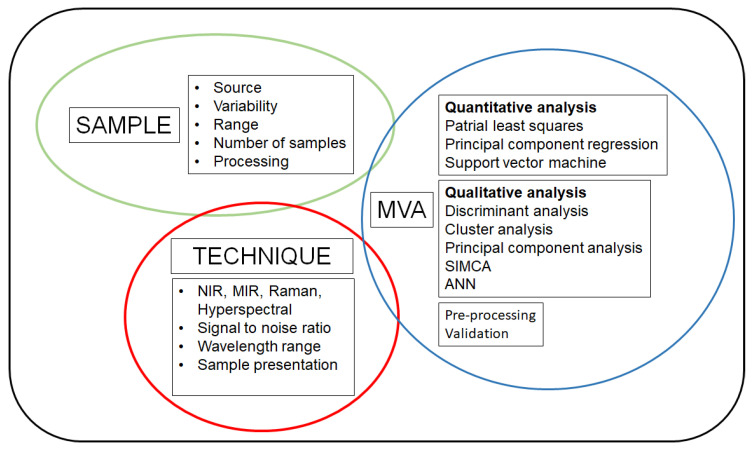
A schematic representation of the main components/variables that affect the sample, technique and data analysis.

These developments require a basic understanding of the different variables that contribute to the system and they include the sample, fundamentals of spectroscopy, data processing and analysis, sampling protocols, and limit of detection (see Figure 3). The adaptation of vibrational spectroscopy to efficiently and reliably contribute to the expansion in the number of applications related to analytical chemistry, process analytical technologies, traceability of food ingredients, and natural products, makes them an ideal set of methodologies towards sustainability along the food value chain. An increasing number of research groups have investigated the use of vibrational spectroscopy, as shown in several applications reported in the literature. However, commercial implementation of these techniques is still under development in some industries.

Even though several articles have been published in the scientific literature, most of them describe feasibility or potential applications of vibrational spectroscopy, where small datasets containing few samples are analysed and cross-validation, rather than an independent dataset, is used to validate the developed models (e.g., calibration). Adding to this is the little in-depth understanding of the reference lab (e.g., standard error of the laboratory method). Most of the application of vibrational spectroscopy are considered correlative methods, and their accuracy depends on the error of the reference method. Therefore, knowledge of the extent to which results are repeatable using wet chemistry or biochemical procedures is of paramount importance in judging the reliability calibration. It is important to remember that the wet chemistry or reference data with all their known inadequacies are used to assess the performance of the calibrations; thus, before assessing the accuracy of a calibration or model, the error associated with the reference method should be known, and this is a fact that is often ignored. The lack of interpretation of loadings, significance of coefficients of regression, and inter-correlations among measured variables and chemical compounds are usually missing from the interpretation.

The use of MVA reveals interesting information about the system but important bits might remain undiscovered. The extent or the use of good MVA (e.g., new algorithms, new software, or mathematical pre-processing) is meaningless if we fail in evaluating the best sample presentation, processing or interactions of the sample collection and analysis.

One of the interesting aspects of the modern integration of these technologies is that it requires and sources information and knowledge from many fields (e.g., spectroscopy, analytical chemistry, data analysis, biology, physics, etc.). This determines the unique multidisciplinary characteristic of this approach. A close collaboration between several researchers is therefore critical for the application and development of the technology. It is also important that everyone involved in the process understands and agrees upon the goals and requirements of the study beforehand to reduce the risk of weak links in the study. The definition of protocols for reporting the outcomes and results of any given study is also important.

Knowing and understanding the reference laboratory method (such as the standard error of the lab method), the limitations of the method, the physics and chemical basis of the spectra, as well as knowing and interpreting the interactions that exist between the sample and the instrument, will allow the user to better interpret the calibration or obtained mathematical relationships. It is therefore important that the individual that developed such calibrations has this knowledge in order to produce a method that can be reliable.

Martens [121] has highlighted that the scientific process of boring into the solid “mountain of the unknown” never stops, and that it is continuous. The author suggested that statistically valid claims must be replicated independently, intuitive hunches should be chased and solid manmade theories should be assessed critically.

The advantages and ability of vibrational spectroscopy to predict multiple parameters and speed of analysis mean that we have a powerful tool that can revolutionise the way we produce foods. The future development of such applications will provide the industry with a very fast and non-destructive method to monitor composition or changes and to detect unwanted problems, providing a rapid means of qualitative rather than quantitative analysis. Moreover, the choice of measuring device(s) may benefit from the experience in, e.g., multichannel diffuse near infrared (NIR) spectroscopy measuring many properties—preferably more than necessary, (it usually does not cost much extra).

However, various hurdles still hinder the growth and development of vibrational spectroscopy applications. Among them is the reluctance to accept the incorporation of vibrational spectroscopy with new statistical tools, such as multivariate data analysis techniques, as routine analytical or quality control methods. Besides, most of the current courses and training programmes in food still focus on the so-called classical approach where several aspects related to the incorporation of new technologies, sensors and programming are not yet incorporated in the curricula. The same can be said regarding research and other aspects of informal training and extension. Together with the silo mentality that still exist in the food industry, this hinders the possibility of exploiting the full potential of these systems by the industry.

Finally, one of the most important and critical aspects of the development of vibrational spectroscopy is the need for an appropriate level of training. For example, although knowledge of the chemistry of a sample material is useful, routine analyses can be performed by analysts with a high-school education. On the other hand, calibration development (interpretation, application and monitoring) is by far the most critical aspect and thus requires a high level of expertise, particularly in multivariate data analysis, in order to make an application successful. Where methods based on vibrational spectroscopy have been applied in industry situations, the potential savings, reduction in time and cost of analysis have been demonstrated. These methods show promising potential for in-field and process analysis.
